# Molecular Determinants of Chronic Venous Disease: A Comprehensive Review

**DOI:** 10.3390/ijms24031928

**Published:** 2023-01-18

**Authors:** Davide Costa, Michele Andreucci, Nicola Ielapi, Giuseppe Filiberto Serraino, Pasquale Mastroroberto, Umberto Marcello Bracale, Raffaele Serra

**Affiliations:** 1 Department of Law, Economics and Sociology, University “Magna Graecia” of Catanzaro, 88100 Catanzaro, Italy; 2 Interuniversity Center of Phlebolymphology (CIFL), International Research and Educational Program in Clinical and Experimental Biotechnology, University “Magna Graecia” of Catanzaro, 88100 Catanzaro, Italy; 3 Department of Health Sciences, University “Magna Graecia” of Catanzaro, 88100 Catanzaro, Italy; 4 Department of Public Health and Infectious Disease, “Sapienza” University of Rome, 00185 Rome, Italy; 5 Department of Experimental and Clinical Medicine, University “Magna Graecia” of Catanzaro, 88100 Catanzaro, Italy; 6 Department of Public Health, University of Naples “Federico II”, 80138 Naples, Italy; 7 Department of Medical and Surgical Sciences, University “Magna Graecia” of Catanzaro, 88100 Catanzaro, Italy

**Keywords:** chronic venous disease, varicose veins, chronic venous leg ulcers, genetics, extracellular matrix, histopathology

## Abstract

Chronic Venous Disease (CVD) refers to several pathological and hemodynamic alterations of the veins of lower limbs causing a wide range of symptoms and signs with a high prevalence in the general population and with disabling consequences in the most severe forms. The etiology and pathophysiology of CVD is complex and multifactorial, involving genetic, proteomic, and cellular mechanisms that result in changes to the venous structure and functions. Expressions of several genes associated with angiogenesis, vascular development, and the regulation of veins are responsible for the susceptibility to CVD. Current evidence shows that several extracellular matrix alterations (ECM) could be identified and in some cases pharmacologically targeted. This review shows the most up to date information on molecular determinants of CVD in order to provide a complete overview of the current knowledge on this topic. In particular, the article explores the genetic influence, the hormonal influence, ECM imbalance, and histopathology of CVD and the role of endothelial dysfunction in CVD.

## 1. Introduction

Chronic Venous Disease (CVD) refers to pathological and hemodynamic alterations of the veins of lower limbs that cause a wide range of symptoms and signs, ranging from mild clinical manifestations such as telangiectasia, reticular veins, and varicose veins (VV) to more severe forms such as skin changes and chronic venous leg ulcers (CVLUs). CVD has a prevalence of up to 57% and 77% in men and women, respectively, in the adult general population, considering the presence of any related clinical manifestation in affected patients [1,2].

In particular, CVD clinical manifestations are described by the clinical, etiological, anatomical, and pathophysiological (CEAP) classification, which defines the following clinical classes: C_0_ includes no visible or palpable signs of venous disease; C_1_ includes telangiectasia and reticular veins; C_2_ includes trunk VV of variable origin; C_2r_ includes recurrent varicose veins; C_3_ includes lower limbs edema; C_4a_ includes pigmentation or eczema; C_4b_ includes lipodermatosclerosis or atrophie blanche; C_4c_ includes corona phlebectatica; C_5_ includes healed ulcer; C_6_ includes active ulcer; and C_6r_ includes recurrent active ulcer. [3] The most severe form of CVD is defined as Chronic Venous Insufficiency (CVI) and corresponds to the C_3_–C_6_ classes of CEAP [1,2].

The explicit mechanisms of CVD are not well understood, but it seems that genetics and several proteomic alterations play an important role in the susceptibility, development, and progression of CVD [4].

The current treatment of CVD may be medical and/or physical and/or surgical, but none of them could be considered definitely effective for patients suffering from CVD. Despite best surgical or endovascular care, recurrence following vein surgery is more than 20%, and about 25–50% of CVLUs remain unhealed after six months of adequate treatment [5,6,7]. Hence, in this area, the current interest of Precision Medicine (PM) for molecular determinants of CVD has increased in order to find molecules that could help to predict this disease and its complications and to better tailor treatment strategies and the related follow-up of patients [8,9,10]. In this comprehensive review, we examine and discuss the most up to date findings surrounding cellular, molecular, and genetic advances in determining CVD, and its progression into its main complications such as CVLUs.

In conducting this review, the searched libraries were Web of Science, Scopus, ScienceDirect and Medline.

The keywords used with various combinations were “chronic venous disease”, “varicose veins”, “venous ulcer”, “genetics”, “proteomics”, “hormone”, “molecular”, “pathophysiology”, and “histopathology”.

## 2. The Genetic Influence

More than 60% of patients with CVD have a clear association among family members; thus, a clear genetic influence exists behind the pathogenesis of CVD [11]. In particular, Cornu-Thenard et al. studied 134 families and found that 90% of children will develop CVD when both parents suffer from this disease, compared with 25% of males and 62% of females when one parent suffers from CVD, and with 20% when neither parent is affected [12].

There are several approaches that have been used to study the genetic influence in CVD. These include heritability analysis through family studies, differential gene expression analysis, and genomic variation studies, either by comparing individual candidate genes or casting the search more widely, with genome wide association studies (GWAS). [13]. The pathogenesis of CVD is a very complex multifactorial process, and even a single gene effect can cause little effective influence, and the impact of a particular polymorphism will depend also on genotype–environment interactions, considering also epigenetic mechanisms, which may even be specific to single patients’ pathophysiology [14].

The expression of several genes associated with angiogenesis, vascular development, and the regulation of vein wall homoeostasis is altered in CVD [15,16].

Considering angiogenesis and vascular development abnormalities, there is robust evidence for a specific role of some genes such as forkhead box protein C2 (*FOXC2*) gene and vascular endothelial growth factor A (*VEGFA*) gene [15].

The *FOXC2* gene, located on chromosome 16q24, encodes a transcription factor that regulates the expression of genes involved in the normal development of the venous and lymphatic systems. In particular, it is required during the early maturation and formation of venous and lymphatic valves [15,17,18,19,20,21]. Moreover, evidence indicates that *FOXC2* overexpression in venous endothelial cells (ECs) may upregulate the expression of Delta Notch pathway-related proteins such as Notch-1 and its ligand delta-like-4 (Dll4) and Hey2, which plays a key role in the development of vascular networks [22,23]. The molecular alterations in Dll4–Hey2 signaling seem also to be associated with smooth muscle cell hypertrophy and hyperplasia in varicose veins [24]. Foxc2 transcription factor also has a role in regulating angiogenesis via the induction of integrin β3 expression. Integrin β3 is a cell adhesion receptor that interacts mainly with extracellular matrix (ECM) components such as fibronectin and vitronectin [25]. Foxc2 transcription factor may also affect the function of CXC chemokine ligand 12 (CXCL12) and its receptor, CXCR4, which are critical for the process of angiogenesis in the vascular system [26].

Several clinical studies have found that mutations in *FOXC2* gene, as well as specific single-nucleotide polymorphisms (SNPs), are strongly associated with CVD [17,27,28,29,30,31].

*VEGFA* gene encodes for vascular endothelial growth factor A (VEGF-A) protein, which is both a critical regulator of angiogenesis and a pivotal factor that is able to maintain the integrity and the functionality of the vessel wall [15,32]. VEGF-A mediates the growth of new blood vessels from pre-existing vessels (angiogenesis) by binding to the cell surface receptors VEGFR1 and VEGFR2, two tyrosine kinases located in ECs of the circulatory system. Increased expression of VEGF-A seems to determine a significant role in CVD pathogenesis as it is able to increases venous wall permeability determining edema and to decrease the tone of the vein wall, which may lead to vein dilation with blood stasis in the lower extremities’ vein system and subsequent venous hypertension development. Moreover, increased expression of VEGF-A may affect ECM remodeling through the imbalance of several proteolytic enzyme synthesis processes such as Matrix Metalloproteinases (MMPs) [33].

The activity of MMPs in ECM is controlled by specific tissue inhibitor of metalloproteinases (TIMPs) in order to maintain the homeostasis and the balance of ECM that supports the integrity of the vessel wall. The expression of several MMPs is increased in patients with CVD. The main cause of this increase is an imbalance between the activities of MMPs and TIMPs, resulting in the breakdown of ECM homeostasis in the vein wall [15]. In particular, Kunt et al. investigated the relationship between MMP-9 and TIMP-2 gene polymorphisms and CVD risk and showed that individuals with the C allele -418G for TIMP-2 were significantly associated with risk of CVD, whereas the individuals with GG genotype had a lower risk for CVD. They did not find statistically significantly difference between the patients with CVD and healthy controls for *MMP9* gene analysis [34]. On the other hand, Xu et al. found that the -1562C/T allele in *MMP9* is a risk factor for CVD and that the *TIMP2* gene polymorphism -418G/C was also associated with CVD [35,36].

Iron overload has been implicated in the pathogenesis of CVD, and in particular *HFE* gene polymorphisms seem to be linked to developing the diseases and also to accelerating the progression to CVLU formation [15,37]. Specifically, C282Y mutation is responsible for an increase in the risk of CVLU by almost seven times [36]. In fact, it is documented that there is an elevated serum concentration of iron in CVD patients compared with healthy controls [15,37,38,39]. Furthermore, iron overload may serve as a further mechanism for MMP hyperexpression leading to CVLU [40].

Abnormalities in the genes encoding enzymes that control homocysteine metabolism, such as Methylenetetrahydrofolate reductase (*MTHFR*) gene, have been shown to predispose to CVD development [15]. In particular, Wilmanns et al. showed a strong influence of genotypes at *MTHFR* c.677C>T and c.1298A>C on the morphological specification of CVD and progression towards complicated disease (CEAP classes C_3_–C_6_) [41].

A recent study postulated that T-helper-17 (Th17) gene expression may influence CVD onset and progression. Th17 cells are a subtype of pro-inflammatory T helper (CD4+) cells, defined by the production of a cytokine signature, of which IL17 represents the progenitor and may be a role in chronic inflammation that characterizes CVD in its progression to the most severe stages [42]. 

Recently, several large-scale GWAS studies were conducted on CVD [10,43,44,45,46,47,48]. These studies contributed to identifying novel genetic variants such as the genes EGF Containing Fibulin Extracellular Matrix Protein 1 (*EFEMP1*), Potassium Voltage-Gated Channel Subfamily H Member 8 (*KCNH8*), and Src Kinase Associated Phosphoprotein 2 (*SKAP2*) involved in CVD susceptibility, mainly acting by the demodulation of certain MMPs and TIMPs that might result in defects in ECM components, causing vein disfunction [44]. The Collagen Type XXVII Alpha 1 Chain (*COL27A1*) gene variant seems to be also related to ECM imbalance in CVD [10]. Some polymorphisms in the complement factor B gene of major histocompatibility complex (*MHC*) *class III* subregion seem to be linked to CVD, probably by triggering chronic inflammation and immune response [45]. In addition, aberrant gene for protein phosphatase 2B regulatory subunit 1 (*PPP3R1*) and aberrant gene for protein Nuclear Factor of Activated T Cells 2 (*NFATC2*) seem to be linked to chronic inflammation and immune response [10].

Mutations in genes for thrombomodulin (*THBD*) and desmuslin (also called synemin) (*SYNM*) seem to promote the development of varicose veins by altering vein function, while the alteration of Piezo 1 protein, due to the mutation of the gene Piezo Type Mechanosensitive Ion Channel Component 1 (*PIEZO1*), seems to result in significant disorganization of the vascular system in maturation phase during angiogenesis [46]. *PIEZO1* gene and another gene linked to CVD, Solute Carrier Family 12 Member 2 (*SLC12A2*), are also involved in the regulation of both cell volume and vascular tone [47].

Interestingly, some genes such as Potassium Inwardly Rectifying Channel Subfamily J Member 2 and 16 (*KCNJ2* and *KCNJ16*) and SRY-Box Transcription Factor 9 *(SOX9*) seem to be related to CVD, but the mechanistic explanation for disease development remains unclear [47]. 

In recent studies, GWAS analysis confirmed well-known loci such as *FOXC2* gene, *HFE C282Y* allele, and *VGFA* variant, as well as a multitude of SNPs worthy of further investigations [10,48], and more interestingly some of these genes, such as *VEGFA*, *COL27A1*, *HFE*, *EFEMP1*, *KCNJ2*, *PPP3R1*, *SLC12A2*, and *NFATC2*, appear also suitable for pharmacological targeting in the near future [10,47]. Moreover, a polygenic risk score analysis may represents a good predicting tool in CVD patients [10]. 

Regarding the mode of inheritance, several modes have been postulated such as autosomal recessive mode of inheritance, but also autosomal dominant-like inheritance was found and, in some cases, also with incomplete penetrance. Furthermore, in the case that only one parent was affected, no correlation was observed between the sex of the affected parent and CVD in the children. However, it was common to observe pedigrees where males and their fathers, but not their mothers, were affected. All these factors seem to exclude a sex-linked pattern of inheritance, thus highlighting the multifactorial nature of CVD inheritance [29,49]. 

The SNP rs1024611 in the monocyte chemoattractant protein 1 (*MCP1*) gene, also known as C-C motif chemokine ligand 2 (*CCL2*), encodes monocyte chemoattractant protein-1 (MCP-1), one of the most important chemokines that guides the movement of several blood cells, such as monocytes-macrophages, basophilic cells, and T lymphocytes, from the circulation to the site of inflammation. Alteration in *MCP1* regulation may enhance the expression of several inflammatory factors and cells triggering the chronic inflammation that characterizes CVD. MCP-1 also affects VSMCs homeostasis, turning them into a proliferative state, thus contributing to CVD development. The association of rs1024611 was significantly found in C_2_ CVD patients [50].

Table 1 lists the most important genetic evidence discussed in this section.

A growing amount of evidence shows that noncoding RNAs, such as microRNAs (miRNAs) and long noncoding RNAs (lncRNAs), which are RNAs not encoding proteins but with regulatory properties of gene expression, may also be used to identify biomarkers in several cardiovascular diseases [51]. In particular, Wei et al. [52] showed that miR-17-5p, miR-129-5p, miR-1297, miR-20b-5p, and miR-33a-3p seem to be related to the onset of CVD, and three lncRNAs, namely AC114730, AC002127, and AC073342, seem to be effective biomarkers of great saphenous vein incompetence among patients with CVD.

Moreover, MicroRNA-199a-5p is able to regulate *FOXC2* to affect VSMCs morphology and functions in the onset of CVD [53]. In addition, miR-202 seems to affect VSMCs causing abnormal proliferation and phenotypic pathological transition in the context of CVD [54].

Furthermore, miR-301a-3p seems to be involved in venous endothelial damage and may be a trigger in the progression of CVD in more severe for such as CVLU, and probably in the near future may also be used as a biomarker towards this complication [55].

lncRNAs and MiRNAs are also linked to *MMP9* gene expression in the development of CVD [56]. In fact, dysregulations of MicroRNA are linked also to a variety of gene expressions in CVD onset and development [57].

## 3. The Hormonal Influence

Sex hormones play a predominant role in CVD onset and pathophysiology, especially considering the influence of gender in this disease (predominance in women). In fact, some studies demonstrate that VVs are associated with increased estrogen receptors (ERs) and progesterone receptors (PRs) expression and with decreased androgen receptors (ARs) expression in all tunica layers of the vein wall [58,59]. Moreover, the expressions of ERs, in particular ERα, ERβ, and G protein-coupled ER (GPER), seem to correlate with the severity of CVD and with the clinical stage of the disease [58]. 

From a mechanistic point of view, ERs act by ER-mediated enhanced venous relaxation and decreased venous contraction, causing more distensible veins. In addition, progesterone inhibits vascular smooth muscles (VSMCs) contraction. The effect is more evident in women, as the estrogen levels are more elevated than in men; nevertheless, the same mechanism is also related to men [58,59,60,61,62].

Moreover, estrogens can induce the migration of VSMCs and induce CVD, also stimulating the promotion of MMP-2 and MMP-9 expression through the classical pathway of ERs [63].

The role of sex hormones could also explain, in part, the significant and statistically strong association between pregnancy and the development of VV during or after this time, according also with the number of pregnancies [64].

## 4. ECM Imbalance and Histopathology of CVD

Several pieces of evidence suggest that the development of CVD is secondary to defects in ECM components, determining weakness and altered venous function with warped valves, a thin vein wall, and subsequent dilated veins. In fact, the ECM provides a structural framework of a wide range of proteins such as collagen, proteoglycans, elastin, glycoproteins, and fibronectin in which various cellular components are embedded. The ECM is a dynamic system that maintains the integrity and homoeostasis of the vein through interactions with cellular components such as the endothelium and VSMCs [16]. 

Homoeostasis of the ECM is regulated by a group of enzymes called metalloproteinases (MPs) and TIMPs. There are several families of MPs, such as MMPs, ADAMs (a disintegrin and metalloproteinases), and ADAMTSs (a disintegrin and metalloproteinases with thrombospondin motifs). Specifically, MMPs are a group of several multi-domain zinc-dependent enzymes that have the ability to influence the migration, proliferation, and apoptosis of VSMCs, ECs, and inflammatory cells; ADAMs are a family of transmembrane and secreted proteins that have functions in cell adhesion and the proteolytic processing of the ectodomains of diverse cell surface receptors and signaling molecules; ADAMTSs descend from the ADAMs, but they have diverse functions and major roles, including the maturation of proproteins such as procollagen and ECM remodeling during morphogenesis. MMP-1, MMP-8, ADAM-17, and ADAMTS-4 appear to be primarily involved with chronic or irreversible complications of CVD such as CVLU. ADAMTS-1 and ADAMTS-7 were found to be elevated in all stages of CVD with respect to the healthy subjects. ADAMTS-5, TIMP-1, and TIMP-2 seem to be negatively associated with the progression of the disease and decrease progressively during the worsening of CVD. MMP-9, especially if complexed to neutrophil gelatinase-associated lipocalin (NGAL), is found to be elevated in all stages of CVD and in particular in more severe cases. NGAL is a protein belonging to the lipocalin family, with the ability to positively modulate the activities of MMP-9, protecting MMP-9 from the proteolytic degradation of TIMPs [65,66,67,68,69]. Moreover, MMP-2 seems also to have the effect of increasing vein wall tension with subsequent venous relaxation [70].

Furthermore, the regulation of the MPs is complex and occurs at different levels, including at gene transcription, protein translation, pro-MP activation, and endogenous inhibition by plasma proteins such as α2-macroglobulin and TIMPs, and thus it is not simple to identify the specific cause of alteration [16].

Interestingly, some evidence has showed that MP alterations could be druggable and that the assumption of some non-selective antagonist of MPs, such as tetracyclines, could results in reducing the time of wound healing in CVLU [71,72].

From a histopathological point of view, the degradation of ECM, due to MPs imbalance, is likely to contribute to the weakening and dilation of the vein vessel wall. In fact, the disruption of the elastic fibers, including fragmentation of the elastic laminas, and the thickening of individual collagen fibers, as well as the imbalance of elastin and collagen content have been found in VV. Furthermore, several changes in the cellular and ECM structure have been identified in all the layers of the vein wall in VV. VSMCs proliferate and infiltrate underneath the endothelial cell (EC) lining, leading to irregular intimal hyperplasia with associated collagen deposits [16,73]. These changes in VSMCs are mainly due to an imbalance between apoptosis and cell proliferation determining irregular proliferation patterns, probably mediated also by anomalous chemical signals such as those coming from inflammatory cytokines that can also be triggered by MPs [65,74] and also from transforming growth factor beta 1 (TGF-β1), a protein that is present in several cells of the cardiovascular system, such as ECs, VSMCs, myofibroblasts, and macrophages and is also a key regulator of ECM synthesis and remodeling. TGF-β1, in advanced stages of CVD, such as skin changes and lipodermatosclerosis (C_4a_ and C_4b_ clinical classes of CEAP), seems to induce a significant dermal fibroblast proliferative response with a subsequent progressive dermal fibrosis, typical of the aforementioned clinical classes of CVD [75].

Moreover, a recent study that evaluated samples obtained from VVs found a decreased vein innervation, and this may also account for the decreased venous contraction capacity of VSMCs, determining also the warping and the separation of valve cusps triggering the venous reflux. Other findings were VEGF-positive structures with an increasing trend in relation to the staging of the disease, and a decreasing expression of protein gene product 9.5 (PGP 9.5) in relation to the staging of the disease. PGP 9.5 is a neuroendocrine cell-specific protein related to vein innervation. Numerous amounts of fibronectin in VVs were found below the endothelial lining, and this accounts for a response to inflammatory processes in damaged veins. MMP-9 structures were also found with increasing trends in relation to the disease staging [76].

Furthermore, other histopathologic alterations have been documented in VVs such as focal intimal discontinuity and the denudation of endothelium, probably due to hypoxic damage to the ECs [77].

The ECM alteration determined by MP imbalance may also have a systemic localization involving other human structures characterized by collagen and elastin metabolism. In particular, the contemporary presence was documented, in selected patient populations, of multiple collagen-related disorders such varicocele, inguinal hernia, and VVs that had also MMP-9 abnormalities at tissue and plasma evaluation in common, confirming that CVD may be related to a more general and progressive disorder of collagen metabolism [78,79].

## 5. The Role of Endothelial Dysfunction in CVD

Chronic inflammation is pivotal in determining CVD and causes localized endothelial activation and subsequent endothelial dysfunction (ED) by reducing the synthesis of anti-inflammatory agents and, on the other hand, enhancing the expression of proinflammatory and prothrombotic molecules [80]. ED is also related to vascular tone and shear stress [81]. Shear stress is also able to influence vessel wall geometry due to several hemodynamic forces [82]. In CVD, shear stress seems to be reduced, and this may promote vein wall and venous valve pathological changes and the activation of ECs and leukocytes, thus enhancing the expression of adhesion molecules and determining the infiltration of inflammatory cells into venous wall and leaflets, thus contributing to chronic inflammation [80]. The activation of ECs determines specific signaling, similarly to innate immunity mechanisms, through different pathways stimulating the production of inflammatory mediators such as chemokines, cytokines, growth factors, and MPs, that further worsen and perpetuate chronic inflammation [80,83,84]. Healthy ECs are influenced by laminar shear stress, which also enhances the expression and the activity of endothelial nitric oxide synthase (NOS), promoting NO production. NO inhibits the proliferation of SMCs and stimulates vasodilatation and has effective anti-inflammatory effects. In areas of disturbed blood flow, such as VVs, a decreased shear stress acting on ECs produces several proinflammatory responses determining the anomalous remodeling of the vein wall [80,85]. 

The vascular endothelial surface is covered by the glycocalyx (GCX) matrix that confers important functions in vascular homeostasis [86]; it is the most luminal layer of the blood vessel, and it extends on and within the vascular wall and is composed mainly of proteoglycans, glycosaminoglycans (GAGs), and plasma or endothelial-derived glycoproteins [87]. Like the functions of the ECM, endothelial glycocalyx regulates several extracellular functions, such as signal transduction, cell adhesion, and localized deposition of growth factors and other signaling molecules. In addition, the glycocalyx also provides some specific functions that include maintaining selective vascular permeability, modulating thrombosis, and regulating leukocyte extravasation [88]. Due to its constant exposure to shear and circulating enzymes such as neuraminidase, heparanase, hyaluronidase, and MPs, the GCX composition has a highly dynamic regulation with constant replacement or re-arrangement of molecules, ranging from shedding or enzymatic degradation to de novo biosynthesis [86,89].

Among GCX glycoproteins, the most relevant are the immunoglobulin superfamily, of which vascular cell adhesion molecule 1 (VCAM-1), intracellular adhesion molecule 1 and 2 (ICAM-1 and -2), and platelet/endothelial cell adhesion molecule 1 (PECAM-1) are well known members related to CVD [90,91,92]. PECAM-1 is also a mechano-transducer that can be stimulated by external forces and that can, as a consequence, initiate specific shear-dependent signaling pathways [93]. Interestingly, a recent study demonstrated the occurrence of endothelial to mesenchymal transition (EndMT) in VVs due to disturbed shear stress [94], which is a major contributor of the endothelial dysfunction related to the development of CVD [95]. EndMT is a dynamic process in which endothelial cells suppress constituent endothelial properties and take on mesenchymal cell behaviors, and in most severe forms, mesenchymal characteristics may become prominent and endothelial functions may significatively diminish [96]. Moreover, inflammatory signaling has been associated with EndMT. ECs respond by expressing cell surface adhesion molecules and secreting proinflammatory factors during the inflammation process, and the reaction is activated by two main cytokines: Tumor Necrosis Factor α (TNF-α) and interleukin β (IL-1β). IL-1β is a proinflammatory cytokine involved in endothelial dysfunction and a key inducer of EndMT. In addition, hypoxia and low-oxygen stress can induce EndMT [97].

Blood cells and endothelial dysfunction are also linked. In particular, leukocyte recruitment on ECs of veins during inflammation is regulated by P-selectin and P-selectin glycoprotein ligand-1 (PSGL-1), which also mediates the interaction between platelets and ECs [98]. Moreover, venous stasis may lead to red blood cell (RBC) extravasation in the surrounding tissues. Then, RBCs are degraded by interstitial macrophages, and released iron from RBCs is stored in the form of ferritin to later produce hemosiderin, which is responsible for the limb pigmentation (lipodermatosclerosis, C_4b_ of CEAP) in patients with CVD. Furthermore, T lymphocytes are also crucial players in the inflammatory reaction occurring in the venous wall and valves of patients with CVD. T lymphocytes could be differentiated into various specific subsets, such as Th1, Th2, Th17, etc. [99].

As previously discussed, MCP-1 also triggers chronic inflammation pathways, recruiting several blood cells, thus contributing to endothelial dysfunction [50,100,101].

From a hemodynamic point of view, venous hypertension, which characterizes CVD, is transmitted from the macrocirculation to the microcirculation by the post-capillary venular system, contributing to increasing endothelial shear stress with further damage to the GCX. Consequently, venous hypertension causes venulo-capillary stasis with an increase in endothelial permeability, with the opening of the intercellular spaces, with the extravasation of liquids from the capillaries, and interstitial flooding that may determine the formation of fibrin caps in the pericapillary space. Fibrin caps reduce exchanges between oxygenated blood and tissues, contributing to the formation of CVLUs [102].

Endothelial dysfunction is druggable and can respond to some pharmacological treatments such as the use of sulodexide or mesoglycan, which have pleiotropic effects on the vascular system including anti-inflammatory, endothelial protective, and vasoregulatory effects [103,104,105]. In this regard, a study showed a preventive effect on varicose vein development after MCP-1 expression suppression [106]. Moreover, there are also some clinical studies that confirmed MCP-1 serum concentration changes after venous invasive procedures and also after venoactive drug treatment, and this may account for its role as an effective biomarker in CVD clinical management [107,108,109,110].

**Figure 1:**
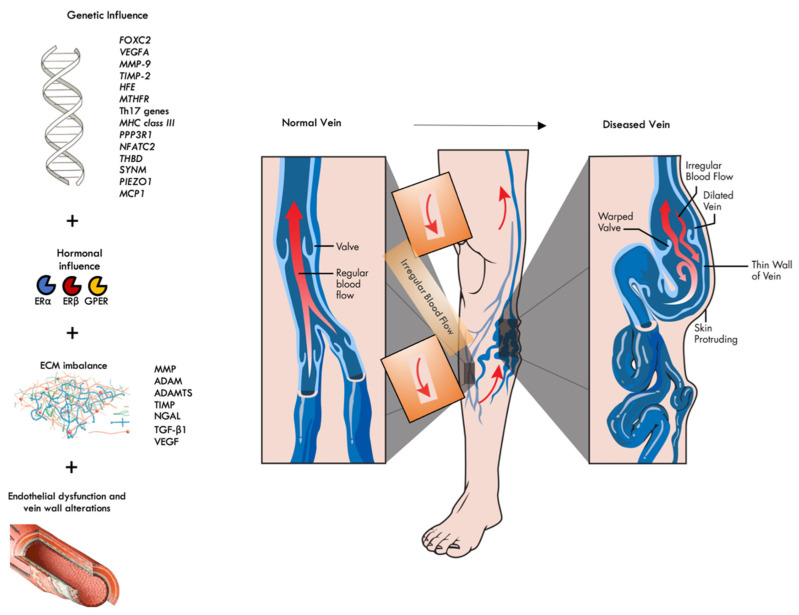
Main pathophysiological mechanisms in CVD development.

Figure 1 summarizes the main pathophysiological steps in CVD development that have been discussed so far in various sections of this article.

*FOXC2*: forkhead box protein C2; *VEGFA*: vascular endothelial growth factor A; *MMP9*: matrix metalloproteinase 9; *TIMP2*: tissue inhibitor of metalloproteinase 2; *MTHFR*: methylenetetrahydrofolate reductase; Th17: T-helper-17; *MHC*: major histocompatibility complex; *PPP3R1*: protein phosphatase 2B regulatory subunit 1; *NFATC2*: Nuclear Factor of Activated T Cells 2; *THB*D: thrombomodulin; *SYNM*: Synemin/desmulin; *PIEZO1*: Piezo Type Mechanosensitive Ion Channel Component 1; *MCP1*: monocyte chemoattractant protein 1; ERα: Estrogen receptor alpha; ERβ: Estrogen receptor beta; GPER: G protein-coupled estrogen receptor; MMP: matrix metalloproteinase; TIMP: Tissue inhibitor of metalloproteinase; ADAM: a disintegrin and metalloproteinases; ADAMTS: a disintegrin and metalloproteinases with thrombospondin motifs; NGAL: neutrophil gelatinase-associated lipocalin; TGF-β1: transforming growth factor beta 1; VEGF: vascular endothelial growth factor.

## 6. Conclusions

Several genetic alterations and genetic polymorphisms can be detected, of which no particular one is able alone to induce susceptibility for the development of CVD, but they are useful in improving our knowledge on the risk factors of this disease. Conversely, the role of ECM and histopathologic alterations is clearer, as well as that of the endothelial dysfunction that underlies the pathophysiological mechanism of CVD; current studies on pharmacological targeting are ongoing, and hopefully in the near future, more tailored options will be available for affected patients.

## Figures and Tables

**Table 1:** Main genes and related implications in CVD development.

Gene Alteration or Polymorphism	Implication in Vascular Development and Angiogenesis	Implication in Vein Wall Integrity and Function	Implication in Chronic Inflammation
*FOXC2*	X	X	
*VEGFA*	X	X	X
*MMP9*		X	X
*TIMP2*		X	X
*HFE*		X	X
*MTHFR*		X	X
Th17 genes			X
*MHC class III*			X
*PPP3R1*			X
*NFATC2*			X
*THBD*		X	
*SYNM*		X	
*PIEZO1*	X	X	
*MCP1*		X	X

CVD: chronic venous disease; *FOXC2*: forkhead box protein C2; *VEGFA*: vascular endothelial growth factor A; MMP9: matrix metalloproteinase 9; TIMP2: tissue inhibitor of metalloproteinase 2; *MTHFR*: methylenetetrahydrofolate reductase; Th17: T-helper-17; *MHC*: major histocompatibility complex; *PPP3R1*: protein phosphatase 2B regulatory subunit 1; *NFATC2*: Nuclear Factor of Activated T Cells 2; *THBD*: thrombomodulin; *SYNM*: Synemin/desmulin; *PIEZO1*: Piezo Type Mechanosensitive Ion Channel Component 1; *MCP1*: monocyte chemoattractant protein 1.

## Data Availability

All data generated or analyzed during this study are included in this published article.
